# Urine PCR Evaluation to Diagnose Pulmonary Tuberculosis

**DOI:** 10.5812/jjm.9311

**Published:** 2014-03-01

**Authors:** Ali Akbar Heydari, Masood Reza Movahhede Danesh, Kiarash Ghazvini

**Affiliations:** 1 Infectious Diseases Preventable by Vaccine Research Center, Mashhad, IR Iran; 2 Infectious Diseases Department, Imam Reza Hospital, Mashhad University of Medical Sciences, Mashhad, IR Iran; 3 Microbiology Department, Imam Reza Hospital, Mashhad University of Medical Sciences, Mashhad, IR Iran

**Keywords:** Tuberculosis, *Mycobacterium tuberculosis*, Urine, Polymerase Chain Reaction, Sensitivity and Specificity

## Abstract

**Background:**

Culture and specific staining (including Zeil-Nelson and fluorescent methods) are standard measures for the diagnosis of tuberculosis (TB). These methods are time-consuming and sometimes have a low level of accuracy. In addition, in some cases obtaining samples for smear and culture involves invasive procedures; while in other cases there is no suitable sample for evaluation. Therefore, there is a need for faster and more accurate diagnostic methods.

**Objectives:**

The current study investigated the diagnostic value of tuberculosis-polymerase chain reaction (TB-PCR) of urine in the diagnosis of pulmonary tuberculosis (PTB).

**Patients and Methods:**

This case-control study included; 77 proven pulmonary tuberculosis cases (according to the national TB protocol), and 30 subjects who were completely healthy. The urine samples (50 mL) were mixed with 0.5 mL Ethylene diamine tetraacetic acid. DNA extraction and PCR testing were performed on all blood samples using SI 6110 primers. *Mycobacterium tuberculosis* was also cultivated in the sputum and urine samples of the patients.

**Results:**

Results of the current study indicated that 48 (62.3%) patients out of 77 had a positive sputum culture. Urine cultures and acid-fast smears were negative. Urine PCR-TB was positive in 48.0% (37/77) of the patients. The speciﬁc TBPCR complex was positive in 56.2% (27/48) of the positive cultures and 34.4% (10/29) of the negative culture PTB patients. The control group had negative urine PCR (sensitivity 56.2% and specificity 100%).

**Conclusions:**

With regard to the ease of urine sample preparation and the 100% specificity the PCR method, performing urine PCR could be used as a diagnostic aid in PTB cases obtaining sputum samples is problematic.

## 1. Background

Every year, nine million individuals develop active tuberculosis (TB) and approximately 1.5 million people die from it (1). According to the World Health Organization in 2010, the prevalence and incidence of TB in Iran was 17 and 13 per thousand, respectively. The incidence has decreased from 21 in 2007, to 17 in 2010 (2). Stained smears and cultures of sputum samples are the first step in evaluating patients with suspected pulmonary tuberculosis (PTB). However, in some cases, there is no sputum, or it is impossible to obtain due to the depressed mental status of the patient, thus it is necessary to search fora faster and/or more accurate method, such as blood or urine TB-polymerase chain reaction (PCR). Furthermore, in some cases obtaining specimens for smear and culture requires invasive procedures. On the other hand, sometimes the patient’s clinical status does not permit the performance of these procedures. Finally, in many cases *Mycobacterium tuberculosis* is not detectable through microscope or culture.

In recent years, molecular diagnostic methods using rapid nucleic acid amplification tests (RNAAT), have replaced traditional diagnostic methods. These methods can detect very small numbers of microorganisms in clinical specimens. In cases of smear-positive specimens, their sensitivity and specificity reaches 95%, while the values for smear-negative cases is 40 - 77%, and more than 95% respectively (3). Considering the previously mentioned points and the fact that urine collection is simple and noninvasive, this method could be a suitable diagnostic tool to diagnose TB.

## 2. Objectives

The current study aimed to evaluate the diagnostic value of PCR to detect *M. tuberculosis* in the urine of patients with PTB.

## 3. Patients and Methods

The study was conducted in the TB Clinic of the Infectious Disease Department at Imam Reza Hospital and the microbiology laboratory of Ghaem Hospital in Mashhad, Iran. The study included 77 outpatients and hospitalized patients with confirmed TB, and 30 healthy control individuals. Out of 77 patients with TB, 37 (48.1%) were male, and 40 (51.9%) were female, and their average age was 51/18 ± 23/54 years. From February 2010 to February 2011, patients who were referred consecutively to the TB Clinic, or were admitted to the Infectious Disease Department of Imam Reza Hospital and diagnosed with (PTB), formed the patients’ group. The control group was selected from outpatients with a diagnosis of other infectious diseases, who did not have a chronic cough, their chest X-ray was normal, and a skin test for TB was negative. The research objectives and characteristics were fully explained to all individuals, and their consent was taken.

The study inclusion criterion for patients with PTB was a confirmed diagnosis of PTB based on the criteria listed in the National Technical Committee on TB (4):

1. Two positive sputum specimen smears,
2. One positive sputum smear specimen, plus a chest radiograph which confirms PTB.

Exclusion criteria were as follows:

1. Patients with evidence of renal TB.
2. Patients who had received more than five days of TB treatment.
3. Patients who had engaged in; risky sexual activity, needle sharing, or other risk factors for HIV acquisition.

The healthy control group was selected from individuals with no history of TB. The first morning urine (50 mL) was collected from both the patient and control groups. A ready-to-use kit QIAGEN (Germany), which is suitable for extracting DNA with a molecular weight higher than 2 µg/mL from urine and other clinical specimens, such as blood, was used to extract the *M. tuberculosis *genome.

Lysate was loaded onto the QIAGEN genomic-tips and when other cellular components were removed from the environment, the DNA was bonded to the column. The obtained specimen was washed again to remove the other remaining contaminants from the environment. The high molecular weight DNA obtained by the mentioned procedures was centrifuged after adding cold isopropanol at 13000 rpm for 5 min. The supernatant was removed from the environment and the DNA plate was washed with 70% cold ethanol. Then the DNA was centrifuged again, and 70% ethanol was removed and the plate was air-dried. The DNA plate was dissolved in 50mL of TE (Tris-EDTA) buffer, which required approximately 150 min to accomplish. The obtained specimens were amplified into 20 µL reaction mixtures by PCR.

**Figure 1.**
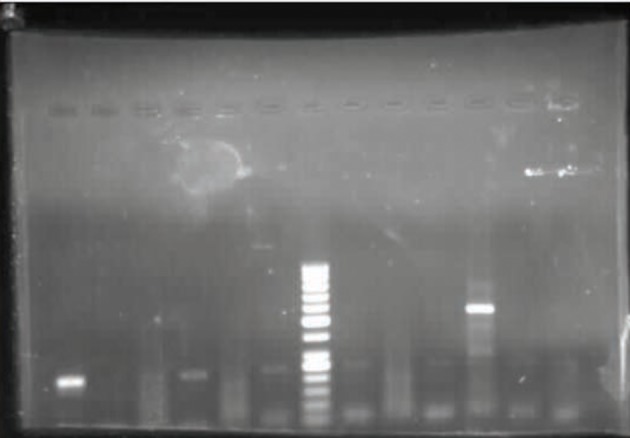
Sample of the Final Products Developed Using Gel Electrophoresis From left to right: Strip 1 - positive control; Strip 2 - negative control; Strip 3-4-5-6 - samples of patients; Strip 7 - ladder 5 0 bp; Strip 8-9-10-11-12-13 samples of patients.

**Figure 2.**
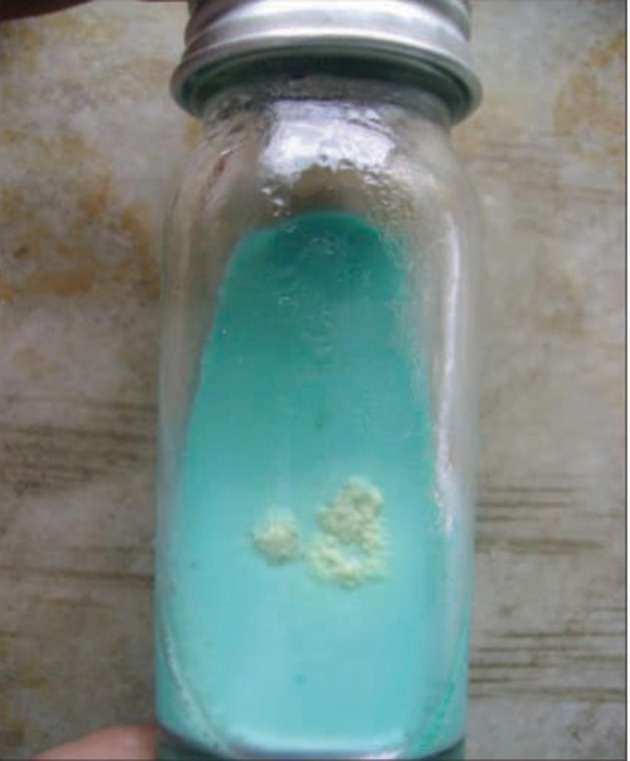
Levenshtein–Jensen Culture Which Was Positive for *Mycobacterium tuberculosis*

Amplification factors, including the initial denaturation process at 94°C for 5 min followed by 32 cycles, each consisting of denaturation at 94˚C for 45 sec, annealing at 54˚C for 60 sec, and extension at 72°C for 60 min, were performed. The final amplified products were identified using gel electrophoresis (1.5% agarose gel with ethidium bromide) (Figure 1). After removal of the natural flora and other bacteria from the sputum and urine samples, they were cultured on Lowenstein –Jensen (Merck-Germany) and incubated at 37°C (Figure 2).

For staining, the urine or sputum samples were purified and 1 to 2 drops were put on a slide and left to dry under the hood. Then they were fixed and stained with Ziehl. The slides were studied under a 100X oil immersion light microscope. Descriptive statistical analyses were used for the data (including demographics, clinical, and results related to blood PCR).

## 4. Results

Out of the 77 patients with TB, 54 were Iranian (70.1%) and 23 were Afghan nationals (29.9%). The sputum cultures were positive in 48 of the 77 patients, and 55.2% of those with negative culture results were male, while in the positive culture results the percentage was 43.8%. The urine smears and cultures were negative in the case group. Urine culture was not done for the control group although all of them were urine PCR negative. Out of 77 patients, 37 patients were urine PCR positive for *M. tuberculosis*, and out of the 48 patients with a positive sputum culture, 27 patients also had positive urine PCR. Out of 29 individuals with negative sputum culture, 19 patients had negative urine PCR. Kappa agreement results showed that there was no agreement between the PCR and the sputum culture results (Table 1). A sensitivity of 56.2% and a specificity of 100%, were found for urine PCR compared to positive sputum smears.

**Table1.** Frequency Distribution of PCR Results on Sputum Culture Results

PCR	Sputum Culture Results		Kappa Agreement
	Positive, No. (%)	Negative, No. (%)	
**Negative**	21 (43.8)	19 (65.5)	0.202
**Positive**	27 (56.2)	10 (34.5)	0.202
**Total**	48 (100)	29 (100)	

## 5. Discussion

A major problem in the diagnosis and treatment of PTB is the paucibacillary form of TB, especially in endemic countries where poverty and the high cost of TB treatment are also problematic (5, 6). There are only a few studies conducted on the use of specimens other than sputum to diagnose PTB in both HIV-positive and HIV-negative cases. Urinary excretion of *M. tuberculosis* was reported for the first time in 1975 by Bentz et al (5). In a study conducted in Iran, urine TB- PCR sensitivity and specificity were 31% and 96%, respectively. The current study evaluated urine samples inpatients with culture-positive PTB, while the other study examined all the samples sent to the laboratory, this factor could have caused differences between the two studies regarding sensitivity (7).

Aceti in his study (8) concluded that in patients with PTB and HIV infections, urine PCR can be a good diagnostic method in individuals with active TB. In Nagasaki`s research in 2002, the Sechi and Githui PCR methods were compared. The sensitivity of the Sechi method was 28.6% and its specificity was 98.4%. The sensitivity of the Githui method was 55.6% and its specificity was 98.4%. The results of the study showed that neither of the methods were sensitive enough to detect tuberculosis (9). The urine PCR was evaluated for diagnostic value in Burkina Faso. The authors of that study concluded that this test is not an appropriate method to detect new TB cases in the normal laboratory tests. However, it can be beneficial for cases where the clinical and bacteriological diagnosis of TB is not definite (10). 

Rebollo in his study collected blood and urine specimens of patients with tuberculosis on several occasions (11). The PCR-TB urine specimens increased the diagnostic sensitivity of blood PCR by 10%. In another study in Italy, researchers determined that the sensitivity of urine PCR was 79%. They concluded that the DNA fragments of TB microorganisms can be measured in the urine of patients with active PTB (12). In a study in India, the PCR of urine samples were positive in 52% of patients with positive sputum cultures and 28.6% of patients with negative sputum cultures, in addition to the 48.5% of patients whose TB was diagnosed by chest radiograph and clinical symptoms. Based on this study, urine culture and PCR can be useful for suspected cases of active tuberculosis, especially in cases where it is difficult to obtain sputum samples (13).

In the current study, PCR-TB was performed on the sample urine of 77 patients with active PTB, who did not have risk factors for HIV acquisition. The sputum culture was positive in 48 (62.3%) patients. The interpretation and management of TB treatment are challenging in situations where there is a negative sputum culture, but a positive sputum smear, particularly if the radiological and clinical symptoms consistent with PTB are not present. The reasons for a positive smear with a negative culture are; non-cultivable *M. tuberculosis*, non-tuberculosis mycobacteria, a long interval between sampling and cultivation, and the susceptibility of the culture medium used. In the current study *Lowenstein –Jensen* medium was used within three days of collection, and the samples were isolated. In some previous studies such as the study in India, there were cases of positive urine cultures; however, in the current study urine cultures were negative. The following reasons can be mentioned for a negative urine culture:

During infection of the lung by *M. tuberculosis*, this organism is swallowed by alveolar macrophages and destroyed. When macrophage cells die, the genomes of the bacteria are released into the plasma and eliminated by the urine (13). Mycobacteriuria is not a continuous process and requires taking repeated samples to detect tubercle bacilli in the urine (13), whereas in the current study only one 50 mL morning urine sample was collected from the patients. In the current study, urine samples for the culture were centrifuged at 3000 rpm, however, if the urine was centrifuged at 10000rpm, the chances of positive results would have increased, as in the study in India (13). The absence of concurrent renal and pulmonary tuberculosis is another reason. In the current study, patients with inflammation of the urinary tract or evidence of tuberculosis of the genitourinary system by physical or sonographic examination were excluded from the study. Perhaps one of the reasons for negative *M. tuberculosis* cultures is the history of using anti-tuberculosis drugs. However, in the current study, patients who had taken the drug for more than five days were excluded. 

Out of the 77 patients; 37 (48%) were positive for TB-PCR, a total of 19 patients were male, they had a median age of 49.6 years, and 25 (67.6%) were Iranian. Their occupation and other demographic variables were not related to the rate of positive urine PCR-TB. Out of the 29 patients with negative sputum cultures, 10 (34.5%) patients were urine-PCR positive. Therefore, the urine PCR helped us to diagnosis TB in 34.5% of patients with negative sputum cultures. In the patients with positive sputum cultures, 56.2% of the cases had positive urine PCR. In the current study, the sensitivity of the PCR was 56.2% and specificity was 100%. The PCR sensitivity in different studies varied from 31% to 79%, and in individuals with HIV, the sensitivity rose to 100%. 

In the current study, smear negative TB patients were not enrolled into the study because of the preference to increase the confidence level of a PTB diagnosis. As a result it could not be determined to what extent urine PCR could be useful in cases of smear-negative tuberculosis. In Singh`s study, higher volumes of urine were used (13), and in Rebollo`s study, the specimens were taken at several different times (11). However, in the current study only 50 mL of the patient’s morning urine was examined, but the sensitivity was higher. There are substances in urine that inhibit PCR, such as; acidic polysaccharides, glycoprotein, urea, and unidentiﬁed non-proteinaceous DNA-associated substances, causing chelation of magnesium ions and possibly preventing PCR amplification (13).

Considering the 100% specificity of urine TB-PCR (despite low sensitivity), it could be useful in the diagnosis of tuberculosis in cases where an adequate sputum sample is not available, or in cases with smear negative PTB. Urine can be used when other clinical specimens have failed to confirm the diagnosis of tuberculosis.
